# Duodenal neuroendocrine cells and neuromedin U in subjects with obesity: Relationship with type 2 diabetes and glucose homeostasis

**DOI:** 10.14814/phy2.70489

**Published:** 2025-08-11

**Authors:** Hassan Bur, Janne Hukkanen, Markku J. Savolainen, Vesa Koivukangas, Tuomo J. Karttunen

**Affiliations:** ^1^ Department of Pathology, Research Unit of Translational Medicine University of Oulu Oulu Finland; ^2^ Department of Pathology Oulu University Hospital Oulu Finland; ^3^ Medical Research Center Oulu Oulu University Hospital and University of Oulu Oulu Finland; ^4^ Research Unit of Biomedicine and Internal Medicine University of Oulu Oulu Finland; ^5^ Department of Surgery Oulu University Hospital Oulu Finland

**Keywords:** chromogranin A, duodenum, GLP‐1, neuroendocrine, neuromedin U, obesity, serotonin, type 2 diabetes

## Abstract

Duodenal endocrine cells contribute to the incretin response, yet their changes in obesity and type 2 diabetes (T2D) remain controversial. We quantified expression of chromogranin A, glucagon‐like peptide‐1 (GLP‐1), serotonin, and neuromedin U (NmU) in duodenal biopsies from 34 participants with obesity (19 with T2D, 15 without) and six healthy controls. Compared to controls, the patients with obesity showed significantly higher proportions of chromogranin A (*p* = 0.007), GLP‐1 (*p* = 0.006), and serotonin (*p* = 0.013) expressing cells, irrespective of T2D status. GLP‐1 expression correlated with HbA1C (*r* = 0.454, *p* = 0.005) and meal test glucose (*r* = 0.455, *p* = 0.0018) but not with insulin. Chromogranin A expression correlated with both GLP‐1 (*r* = 0.486, *p* = 0.003) and serotonin (*r* = 0.475, *p* = 0.003), as well as BMI (*r* = 0.429, *p* = 0.007). NmU was detected in the lamina propria but did not differ among groups. For NmU, an association with insulin (incremental AUC, *r* = 0.363, *p* = 0.045) was observed. In conclusion, obesity was associated with hyperplasia of duodenal GLP‐1, serotonin, and chromogranin A cells. The link between GLP‐1 expression, HbA1C, and meal test glucose indicates that, despite increased numbers of GLP‐1–producing cells, individuals with obesity may still experience insufficient incretin action. No evidence supported a role for NmU as a human decretin in these patients.

## 1 INTRODUCTION

It has been estimated that worldwide about 1 billion people are affected by obesity (Lobstein et al., 2023). The worldwide prevalence of type 2 diabetes (T2D) has more than doubled in the past 40 years, and it is expected to rise up to 10.2% (578 million) by 2030 (Saeedi et al., 2019). The most successful treatment for obesity and T2D is bariatric surgery (Sjöström, 2008).

Gut endocrine cells are implicated in the regulation of metabolism and shown to have a role in the development of obesity and T2D (Nauck et al., 1986). It is known that oral glucose produces a significantly greater insulin response than detected after intravenous glucose exposure, indicating that factors in the gut actively enhance the β‐cell response to glucose. These factors were eventually identified as incretin hormones (Nauck et al., 1986). Glucagon‐like peptide‐1 (GLP‐1) is considered one of the main incretins responsible for augmenting glucose‐dependent insulin secretion in response to nutrient intake (Holst, 2024; Nauck et al., 1986). Fasting GLP‐1 plasma levels are known to be lower in T2D patients compared to healthy individuals (Calanna et al., 2013); however, meta‐analyses indicate that GLP‐1 response to nutrient stimuli is not reduced in most T2D patients (Knop et al., 2012; Watkins et al., 2023). Obesity, even without T2D, has been shown to associate with an abnormal incretin effect (Nauck et al., 2011). However, meta‐analyses suggest that although there is a trend for lower postprandial GLP‐1 levels in obesity as compared with normal weight controls, no significant differences are present in either basal or postprandial levels (Calanna et al., 2013; Knop et al., 2012; Nauck et al., 2011; Watkins et al., 2023). More recent analyses have shown that obesity is associated with a decreased GLP‐1 secretion regardless of glucose tolerance status, with GLP‐1 responses showing an inverse association with obesity (Faerch et al., 2015; Holst, 2024).

GLP‐1 is secreted by intestinal L‐cells (Theodorakis et al., 2006), the numerical density of which is higher in the distal small intestine and large intestine as compared to the duodenum (Guedes et al., 2015; Jorsal et al., 2018). Whether the numbers of intestinal GLP‐1 expressing cells are affected by T2D or obesity is a matter of controversy. Previous observations of small intestinal chromogranin A, GLP‐1, and serotonin expression in obesity and T2D have been collected in Table 1. In T2D, the numbers of duodenal GLP‐1 expressing cells have been reported to be increased by about 42% in a series where body mass index (BMI) in the patients with T2D was about 20% higher than in the controls (Theodorakis et al., 2006). Similarly, Jorsal et al. (Jorsal et al., 2018) found about a 20% higher count of GLP‐1 expressing cell numbers in comparison of controls and patients with T2D with a similar BMI, but the difference was not statistically significant (Jorsal et al., 2018). In the jejunum, Palha et al. did not find a difference between T2D patients and controls, both living with obesity, in the ratio of jejunal GLP‐1 and chromogranin positive cells but did not report numbers of GLP‐1 positive cells (Palha et al., 2018). Osinski et al. (2021) found, in patients with obesity, a decrease of jejunal GLP‐1 secreting cell numbers of about 44% in patients with T2D as compared with patients without T2D, but the difference with normal weight controls was not significant. Wölnerhanssen et al. found that morbid obesity is associated with about a 59% lower number of duodenal GLP‐1 expressing cells and about a 40% lower number of chromogranin positive neuroendocrine cells (Wölnerhanssen et al., 2017). Little et al. found that the numbers of GLP‐1 expressing cells show a negative correlation with BMI (Little et al., 2014). In the ileum and colon, the numbers of GLP‐1 expressing cells were similar in controls and patients with T2D (Jorsal et al., 2018). Thus, no conclusions about quantitative changes in the numbers of GLP‐1 expressing cells in T2D or obesity can be made, and it is not clear whether the alteration of the numbers of intestinal GLP‐1 producing cells explains the reduced incretin effect in some patients with T2D or obesity.

**TABLE 1:** Human studies comparing numbers of small intestinal chromogranin A, GLP‐1 and serotonin expressing cells in healthy subjects and in those with obesity with or without T2D.

Protein	References	Location of samples	Cell counting method	Cell count in healthy controls	Cell count in obesity (no T2D)	Cell count differen‐ce: Obesity vs. controls	Cell count in T2D	Cell count difference T2D vs. controls (significance)	Cell count difference T2D vs. obese without T2D	Cor‐relation of BMI and GLP‐1	BMI in healthy controls (*n*)	BMI in subjects with overweight (*n*)	BMI in subjects with obesity (*n*)	BMI in subjects with T2D (*n*)
Chromogranin A														
Wölnerhanssen et al. (2017)		Duodenum	Cells/areal unit	692 ± 47	413 ± 22	−40%					22.8 ± 0.6 (*n* = 24)		47.2 ± 1.7 (*n* = 27)	
Jorsal et al. (2018)		Small intestine^a^	Cells/epith. area	231/mm^2^ (median)			160/mm^2^	−31% (*p* = 0.006)^b^			27.1 (20.3–30.8) mean (range); (*n* = 12)			26.8 (23.7–31.5) mean (range) (*n* = 12)
GLP‐1														
Theodorakis et al. (2006)		Duodenum	Cells/1000 stained cells	15 ± 1			26 ± 2	42% (*p* = 0.001)			24 ± 3 (*n* = 14)			29 ± 3 (*n* = 17)
Wölnerhanssen et al. (2017)		Duodenum	Cells/areal unit	69 ± 6	28 ± 3	−0.59 (*p* = 0.001)					22.8 ± 0.6 (*n* = 24)		47.2 ± 1.7 (*n* = 27)	
Jorsal et al. (2018)		Small intestine^a^	Cells/epith. area	15/mm^2^ (median)			18/mm^3^	20% (*p* = NS)^b^			27.1 (20.3–30.8) mean (range); (*n* = 12)			26.8 (23.7–31.5) mean (range) (*n* = 12)
Osinski et al. (2021)		Jejunum	Cells/mm mucosa	70^c^	45^c^	−36% (*p* = NS)	25^c^	−64% (*p* = NS)	−44% (*p* = 0.039)		22.7 ± 0.5 (*n* = 22)		46.5 ± 1 (*n* = 21)	43.9 ± 2 (*n* = 21)
Palha et al. (2018)		Jejunum	Area (%) of pos. staining	^d^									39.32 ± 1.04	40.7 ± 1.56
Little et al. (2014)		Duodenum	Cells/mm^2^ mucosa	Not shown						−0.54 (*p* = 0.03)	22 ± 1 (*n* = 9)	28 ± 1 (*n* = 6)	49 ± 5 (*n* = 7)	
Serotonin														
Young et al. (2018)		Duodenum	Cells/mm^2^ mucosa	103 ± 17	186 ± 28	80% (*p* = 0.022)				^e^	25 ± 1 (*n* = 10)		46 ± 4 (*n* = 14)	

^a^ Samples from duodenum to ileum.

^b^ In comparison of all small intestinal measurements.

^c^ Extrapolated from the graph (Figure 3).

^d^ Only GLP‐1/chromogr. ratio shown, no GLP‐1 or chromogranin counts.

^e^ No statistics provided.

Chromogranin A is a structural protein of secretory granules of neurosecretory cells, and a well‐documented marker of intestinal neuroendocrine cells (Facer et al., 1985; Mouland et al., 1994). In addition, chromogranin A and derived peptides have regulatory roles in metabolism (Garg et al., 2023). Many, but not all, GLP‐1 expressing cells are positive for chromogranin A (Jorsal et al., 2018; Kampmann et al., 2016). In T2D, the number of intestinal chromogranin A expressing cells has been reported to be decreased (Jorsal et al., 2018), and a similar decrease has been reported for obesity (Table 1) (Wölnerhanssen et al., 2017).

Serotonin (5‐HT) is an important product of intestinal endocrine cells of the enterochromaffin type. In humans, intraduodenal glucose infusion induces serotonin secretion, and the response is increased in subjects with obesity. Furthermore, the numbers of duodenal serotonin‐secreting cells are increased in obesity (Table 1) (Young et al., 2018). Serotonin has several biological effects, including promotion of gut motility, nutrient absorption, insulin secretion, and lipogenesis (Yabut et al., 2019). There are no studies comparing serotonin‐secreting cell numbers between nondiabetic and diabetic patients with obesity.

Decretins are factors that suppress insulin secretion during fasting. Based on murine experiments, neuromedin U (NmU) was suggested to function as a decretin (Alfa et al., 2015), but further experiments have revealed that NmU has no such role in murine glucose homeostasis (Kuhre et al., 2019; Nakashima et al., 2010; Teranishi & Hanada, 2021). In the murine intestine, NmU is expressed in neural fibers of the lamina propria of the intestinal mucosa and the deeper ganglions and fibers (Augood et al., 1988). However, there are no reports of immunohistochemical expression patterns of NmU in the human intestine and no data about relationships of such expression and obesity or T2D.

Duodenum is the first intestinal segment to receive food and is shown to be critical in the early incretin response, producing the initial increase of the GLP‐1 levels. Indicating the importance of the duodenal incretin response, the early response has been most constantly disturbed in early T2D, and the aberration of the early response in OGTT is a better predictor of risk development of T2D than the late response (Bergman et al., 2024). So far, only a few quantitative analyses of duodenal endocrine cells in patients with T2D and obesity have been published (Jorsal et al., 2018; Little et al., 2014; Palha et al., 2018; Wölnerhanssen et al., 2017). Due to limited case series and discrepant findings, no conclusions about their possible quantitative changes can be made. Patients with more severe obesity (class 2 or 3; BMI >35) have only rarely been studied. Therefore, analyses of endocrine cells in such patient populations are called for. In the present study, our goal was to analyze quantitative signs of duodenal endocrine dysfunction associated with obesity and compare patients with and without T2D. We characterized the overall duodenal endocrine cell population by using chromogranin A as a marker and focused on endocrine factors related to the incretin effect (GLP‐1) and other factors with suspected roles in obesity and metabolism (serotonin). The rationale for studying these three markers included, besides all of them having their own roles in the regulation of metabolism, getting an impression of overall numerical changes of duodenal neuroendocrine cells in obesity and T2D. Although previous findings do not allow any plausible hypothesis to be presented about the quantitative changes of the cells, we hypothesized detecting increased numbers of the cells, as hyperplasia is a frequent response in neuroendocrine cells targeted to increased functional demand (Sachdeva & Stoffers, 2009). Finally, the role of NmU in the human duodenum is unknown, although there is experimental evidence for a role in the regulation of glucose homeostasis.

## 2 PATIENTS AND METHODS

This study is based on a cohort of 34 patients with obesity and a clinically indicated need for bariatric surgery in Oulu University Hospital, Finland, during the years 2010–2015. Of the patients, 19 had been diagnosed with T2D, but no data on the duration of the disease was available. Of the patients, 15 did not have a diagnosis of T2D. For comparison, six healthy, nondiabetic individuals with BMI <30 kg/m^2^ were recruited from subjects undergoing routine gastroscopy for a non‐chronic benign disease. All subjects gave written permission to take part in the study, and the Ethics Committee of the Northern Ostrobothnia Hospital District approved the study design. Details of the study protocol have been published earlier (Härma et al., 2021; Istomin et al., 2022). Patient evaluation included registration of current medication and determination of body mass index. Laboratory investigations included a 2‐h liquid meal test with determination of plasma glucose and serum insulin concentrations (time points at 0, 30, 60, and 120 min), performed after a minimum of 10 h fasting. Total and incremental areas under the curve (AUC) were calculated for both. Fasting glycosylated hemoglobin (HbA1c) concentration was measured.

### 2.1 Tissue specimens and immunohistochemistry

Gastroscopies were performed in a separate visit before the liquid meal test for all subjects, and no significant pathologies were found. During gastroscopy, a minimum of two biopsy specimens from the descending part of the duodenum were taken. Specimens were fixed in buffered formalin and embedded in paraffin with orientation so that vertical sections could be obtained. For basic histology, sections were stained with hematoxylin and eosin. For immunohistochemical staining of chromogranin A, serotonin, GLP‐1, and NmU, sections were deparaffinized, subjected to high‐temperature antigen retrieval, and treated with specific antibodies as described in Table 2. According to the manufacturer, the NmU antibodies have been affinity purified with the antigen and validated with Western blots with lysates of positive and negative cell lines and with protein arrays. In addition, the presence of positive immunohistochemical staining from several human tissues was shown to correlate with mRNA expression (Garczyk et al., 2017). For each antibody, one section from each patient was stained. For the detection of bound antibodies, polymer‐based detection reagents composed of a polymer core linked with a secondary antibody and peroxidase enzyme were used as specified in Table 2. Color was developed with diaminobenzidine. For positive control staining, pancreatic tissue with Langerhans islets was used for chromogranin A and GLP‐1, and gastric antral specimens with their G cells present in most samples served as an additional positive control in chromogranin A staining. Ganglion cells of submucosal autonomic nerve ganglion cells served as positive controls in NmU staining, and for serotonin, lamina propria mast cells were used as positive internal controls. For negative control staining, primary antibodies were replaced with saline or irrelevant antibody.

**TABLE 2:** Antibodies used in immunohistochemical stainings, section pretreatment, antibody dilution, and detection of the bound antibodies. Detection kit specification includes specificity of the secondary antibodies in the kit.

Antigen	Antibody type	Manufacturer	Clone or supplier's code	Pretreatment	Antibody dilution/incub. Time	Detection kit (suppliers code)/manufacturer
Chromogranin A	Mouse monoclonal	Sigma Aldrich/Merck KGaA, Darmstad, Germany	LK2H10	Boiling in tris/EDTA buffer	1:500/30 min RT	Envision Mouse/Rabbit/ (K5007) Agilent, Santa Clara, CA, USA
Serotonin	Mouse monoclonal	Agilent, Santa Clara, CA, USA	5HT‐H209	Boiling in citrate buffer	1:200/60 min RT	Ultravision Mouse/Rabbit (TL‐060‐HD) Thermo Fischer Scientific, Fremont, CA, USA
GLP‐1	Rabbit polyclonal	Abcam, Cambridge, UK	ab22625	Boiling in tris/EDTA, 2 min 800 w + 15 min 300 w	1:2000/60 min RT	Envision Flex/Mouse/Rabbit (K8023)/Agilent
Neuromedin U	Rabbit polyclonal, affinity purified	Sigma Aldrich/ Merck KGaA	HPA025926	Bond Epitope Retrieval Solution 1, Leica Biosystems, Deer Park, IL, USA	1:300/3 h RT	Bond Polymer Refine, Mouse/Rabbit (DS9800)/Leica Biosystems

### 2.2 Quantitation of immunohistochemical stainings

The evaluation of immunostainings was performed by an experienced pathologist (TJK) together with another investigator (HB). For analysis, stained sections were scanned with a Leica Aperio AT2 scanner (Leica Biosystems, Deer Park, IL, USA), and assessed with the program QuPath (version 0.3.0), an open‐source software for digital pathology image analysis (Bankhead et al., 2017). Evaluators were blinded to the subject group and any clinical data.

Vertically sectioned parts of the specimens showing full thickness of mucosa with well‐oriented villi and crypts were used for quantification. Quantification of positive epithelial cells stained for chromogranin A, serotonin, and GLP‐1 was based on the cell detection option of Qupath. Firstly, the cell classifier option of Qupath was trained with annotating villus and crypt epithelium and lamina propria. Performance of the trained classifier was visually confirmed and used for identification and quantification of negative and positive epithelial cells in villus and crypt epithelium. All available vertically cut villi and crypts were included, aiming to get an equal amount of both mucosal components for analysis in each case. Results were provided as a percentage of positive cells of all epithelial cells in duodenal villus and crypt epithelium. For quantification of NmU staining, the pixel classifier option of Qupath was first used for the lineation of mucosa, including both villous and crypt zones. Secondly, a thresholder was created to recognize areas in lamina propria stained for NmU, and the proportion of positive staining of all mucosal area was recorded for each case. Villus and crypt heights were measured from the vertically sectioned parts of the sections with Qupath, and the villus/crypt ratio was calculated for each case.

### 2.3 Statistical analyses

According to tests for normality, observations on subjects with T2D showed non‐normal distributions. Therefore, nonparametric tests were utilized to assess correlations between variables and comparisons between the groups. To compare differences between the controls and all patients, the Mann–Whitney U test was used. In the comparison of controls, patients without T2D and with T2D, the Kruskal–Wallis test was used followed by post hoc pairwise comparisons between the groups using Dunn's test. Correlation analyses between each biomarker (chromogranin A, GLP‐1, serotonin, and NmU) and multiple clinical parameters, including blood glucose and insulin measurements, were performed using the nonparametric Spearman's correlation test. GraphPad Prism (GraphPad Software, La Jolla, CA, USA) was used to calculate the area‐under‐the‐curves (AUC) of the glucose and insulin data with the trapezoidal method during the 2‐h liquid meal test. The calculations of incremental AUCs had measurements performed in the fasting state as a baseline. If the subtraction of the baseline from any subsequent meal test time point resulted in a negative peak in incremental AUC calculation, it was subtracted from the positive peak area (net area calculation).

To account for multiple comparisons in the analyses, adjustments for multiple testing were applied. However, given the exploratory nature of the study and the number of statistical tests performed, adjustment for multiple comparisons was applied in a targeted manner to reduce the risk of false positive findings while maintaining sensitivity to detect potential associations of interest. The resulting *p* values from the Dunn's test were adjusted using the Benjamini–Hochberg false discovery rate (FDR) correction method with a false discovery rate set to 0.05. For the Benjamini–Hochberg test, we used the Excel sheet available in the Handbook of Biological Statistics (McDonald, n.d.). Similarly, in correlation tests for each neuroendocrine marker, Spearman correlation *p* values were adjusted for multiple testing with the Benjamini–Hochberg method. Results were considered significant if the two‐sided *p* value was <0.05. Statistical analyses were conducted with IBM SPSS Statistics 29.0 software (IBM, Armonk, NY, USA).

For adjustment of correlation tests, families of tests were defined based on the structure and purpose of the analyses. Correlations between clinical parameters (BMI, blood glucose, HbA1C, and insulin concentrations) and the biomarker levels (duodenal neuroendocrine cell levels) were analyzed separately for each biomarker, as the biomarkers were here considered unrelated, and the analyses were exploratory in nature. For each biomarker, all correlations with clinical measurements, such as glucose and insulin obtained at multiple time points, as well as the corresponding area under the curve (AUC) values summarizing the longitudinal data, were considered part of a single family of tests. BMI and HbA1C were considered independent and analyzed without adjustment. Adjustments for multiple comparisons were performed separately within the analyses of the full cohort and within each subgroup, as these were considered independent analyses addressing distinct clinical questions.

## 3 RESULTS

Basic demographic and clinical data of patients and controls is summarized in Table 3. As expected, weight and HbA1c were significantly higher for the patients evaluated for bariatric surgery as compared to the control group (*p* < 0.001, *p* = 0.023, respectively; Mann–Whitney *U* test), and in patients with T2D these values tended to be higher in comparison with patients without T2D (*p* = 0.072; *p* = 0.001). There were three patients fulfilling the criteria of prediabetes (American Diabetes Association Professional Practice Committee, 2022) among patients without T2D. There were 18 patients treated with metformin, 17 in the T2D group and one among the patients classified as non‐T2D. Nine patients were treated with dipeptidyl peptidase inhibitor, five with thiazolidinedione, two with sulfonylurea, and one with GLP‐1 analog. No patient was treated with insulin.

**TABLE 3:** Basic data of the patients and controls including duodenal villus and crypt height measurements. Mean and SD are shown.

Variable	Patients			Controls
	All patients	T2D	No T2D	
*N*	34	19	15	6
Gender (M/F)	11/23	7/12	4/11	1/5
Age (years), mean (SD)	46.7 (8.8)	49.0 (8.1)	43.9 (9.0)	50.0 (13.8)
BMI (kg/m^2^), mean (SD)	44.3 (5.4)	41.8 (4.0)	47.4 (5.5)	23.3 (2.6)
HbA1c (mmol/mol), mean (SD)	42.4 (6.4)	46.0 (6.0)	37.9 (3.2)	36.2 (4.7)
Duodenal villus height μm (SD)	432 (63)	444 (70)	418 (55)	380 (58)
Duodenal crypt height μm (SD)	151 (17)	149 (17)	153 (17)	127 (24)
Duodenal villus/crypt ratio	2.9 (0.45)	3.0 (0.47)	2.7 (0.38)	3.1 (0.71)

**FIGURE 1:**
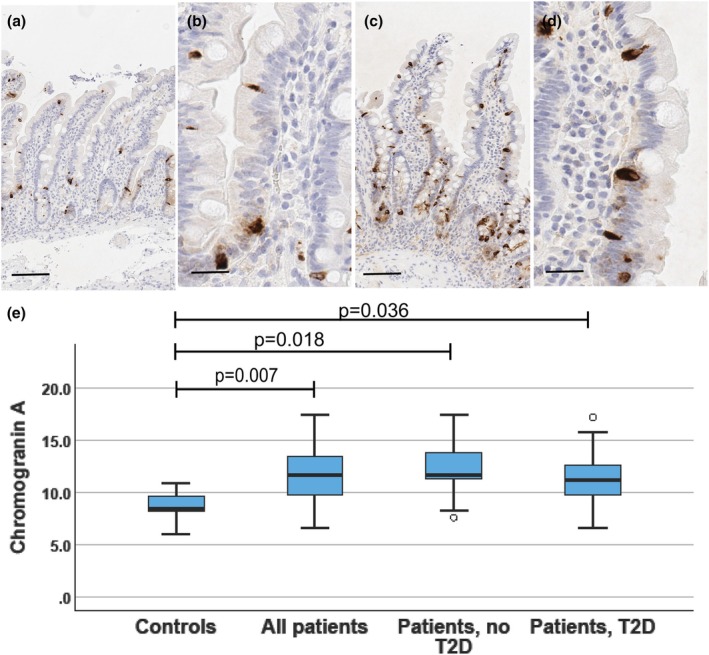
Chromogranin A. Photomicrographs from duodenal biopsies stained for chromogranin A (a, b: Control subject; c, d: Patient with T2D), and a boxblot (e) showing the distribution of duodenal chromogranin A expressing cell numbers. Photomicrographs with a low (a, c; reference line = 100 μm) and a high (b, d; reference line = 20 μm) magnification are shown. Positively stained cells (dark brown) are seen within crypt and villus epithelium. In the boxplot, horizontal lines indicate median, boxes 75% percentiles, and whiskers show the range. The controls and all patients were compared with the Mann–Whitney test. The controls, patients without and with T2D were compared with the Kruskal–Wallis test followed by groupwise post hoc analysis with the Dunn's tests, and adjustment for multiple comparisons with the Benjamini–Hochberg procedure.

**FIGURE 2:**
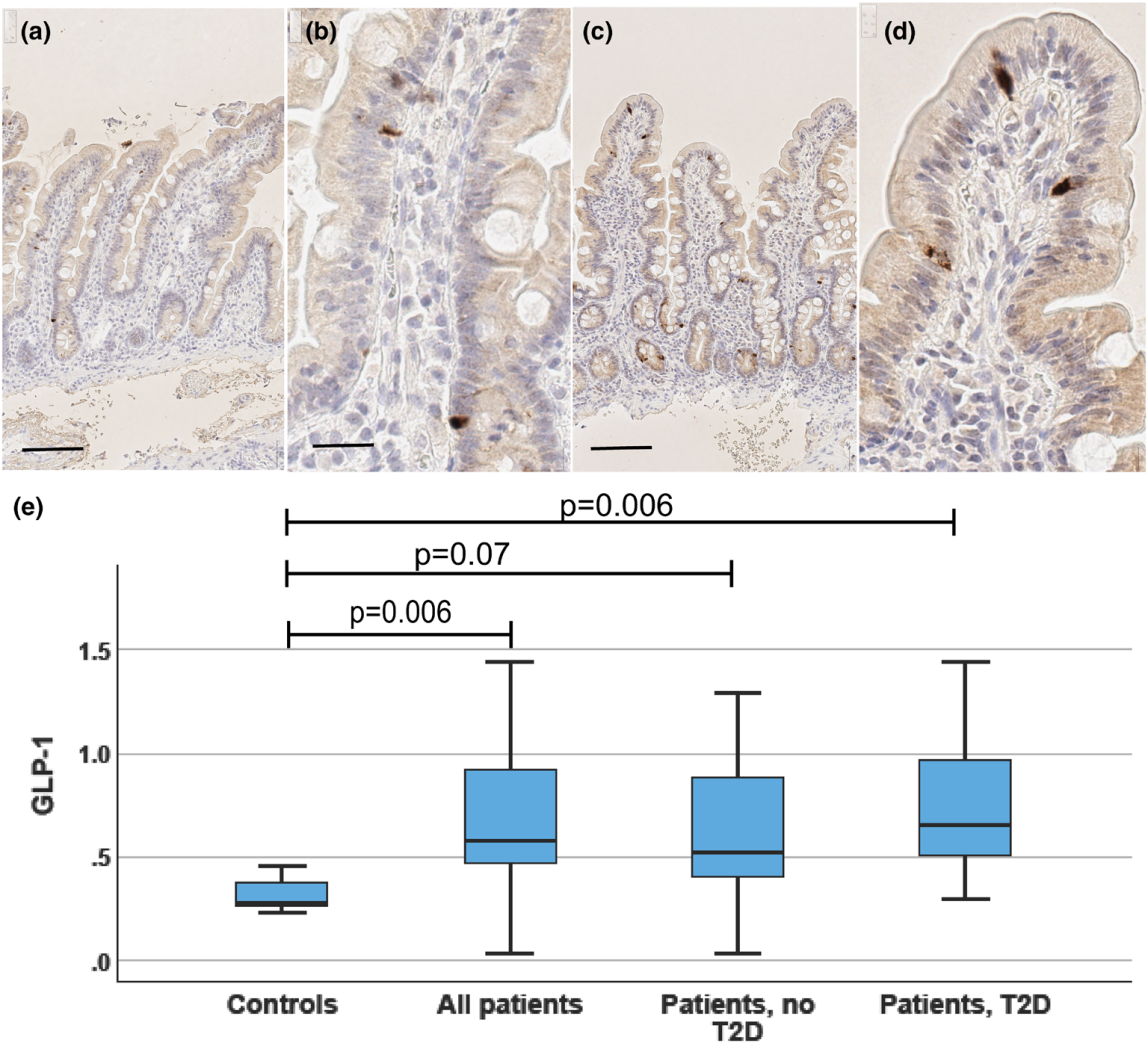
GLP‐1. Photomicrographs from duodenal biopsies stained for GLP‐1 (a, b: Control subject; c, d: Patient with T2D), and a boxblot (e) showing the distribution of duodenal GLP‐1 expressing cell numbers (proportion of all epithelial cells). Photomicrographs with a low (a, c; reference line = 100 μm) and a high (b, d; reference line = 20 μm) magnification are shown. Positively stained cells (dark brown) are seen within crypt and villus epithelium. In the boxplot, horizontal lines indicate median, boxes 75% percentiles, and whiskers show the range. Controls and all patients were compared with the Mann–Whitney test. The controls, patients without and with T2D were compared with the Kruskal–Wallis test followed by groupwise post hoc analysis with the Dunn's tests, and adjustment for multiple comparisons with the Benjamini–Hochberg procedure.

**FIGURE 3:**
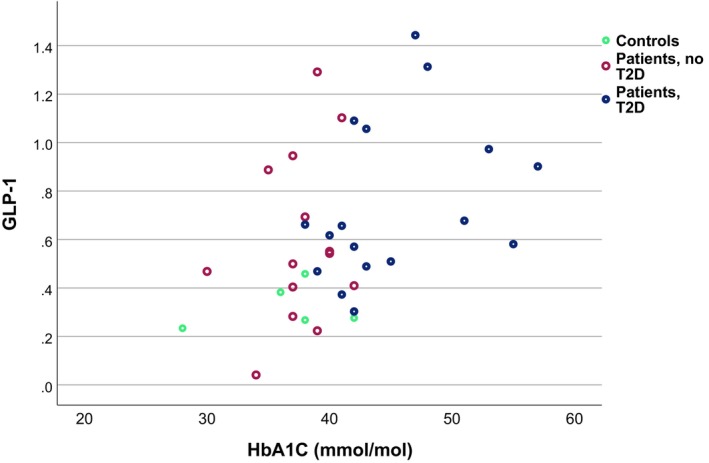
Scatterplot showing relationship between numbers (proportion of all epithelial cells) of duodenal GLP‐1 expressing cells and the concentration of glycosylated hemoglobin (HbA1C; Spearman rank correlation; all subjects *r* = 0.460, *p* = 0.004; patients *r* = 0.443, *p* = 0.015; T2D patients *r* = 580, *p* = 0.014).

**FIGURE 4:**
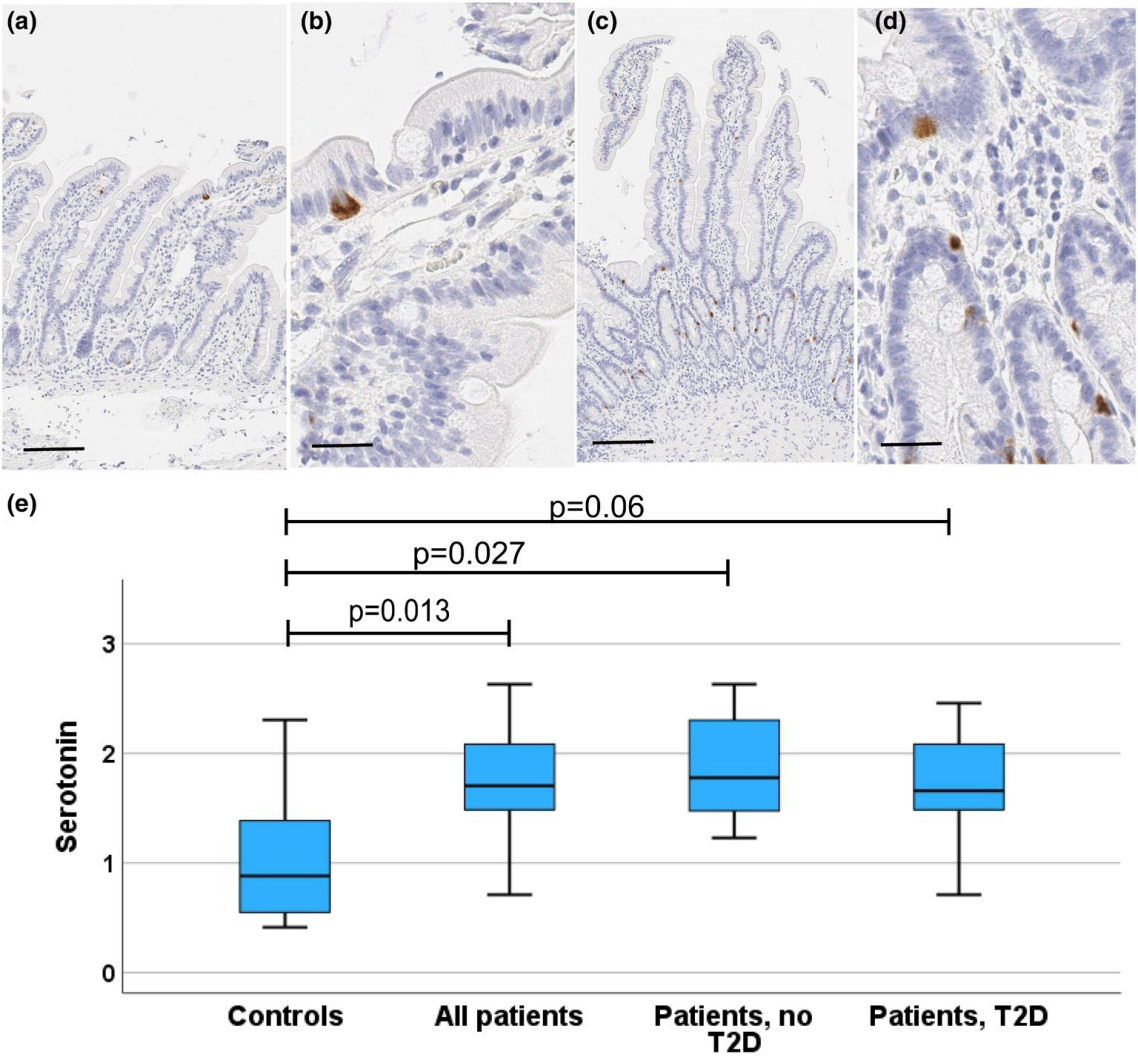
Serotonin. Photomicrographs from duodenal biopsies stained for serotonin (a, b: Control subject; c, d: Patient with T2D), and a boxblot (e) showing the distribution of duodenal serotonin expressing cell numbers (proportion of all epithelial cells). Photomicrographs with a low (a, c; reference line = 100 μm) and a high (b, d; reference line = 20 μm) magnification are shown. Positively stained cells (dark brown) are seen within crypt and villus epithelium. In the boxplot, horizontal lines indicate median, boxes 75% percentiles, and whiskers show the range. Controls and all patients were compared with the Mann–Whitney test. The controls, patients without and with T2D were compared with the Kruskal–Wallis test followed by groupwise post hoc analysis with the Dunn's tests, and adjustment for multiple comparisons with the Benjamini–Hochberg procedure.

No structural abnormalities were seen in the duodenal biopsies. Villus and crypt height and villus/crypt ratio were within the normal range (Table 3) with no significant differences between the groups. Examples of immunohistochemical stainings are shown in Figures 1, 2, 3, 4. Negative control stainings (omission of primary antibody, irrelevant antibody) were all negative, and positive control tissues together with internal positive control cells as described in Materials and Methods showing positive staining confirmed adequate staining quality.

### 3.1 Chromogranin A

Chromogranin A expression was present in a subpopulation (about 11%) of villus and crypt epithelial cells (Figure 1). The proportion of positive cells of all mucosal epithelial cells was significantly higher in patients as compared to controls (controls vs. all patients *p* = 0.007; controls vs. non‐T2D patients, *p* = 0.018; controls vs. T2D patients, *p* = 0.036; Figure 1). However, there was no difference between patients with or without T2D (*p* = 0.405) and neither did sex affect the chromogranin A expression. The proportion of chromogranin‐expressing cells showed a significant correlation with BMI among all subjects (*r* = 0.429, *p* = 0.007; Spearman correlation), but there was no association with meal test glucose concentrations (AUC *r* = 0.244; *p* = 0.152; incremental *r* = 0.274; *p* = 0.106) or insulin response (AUC *r* = 0.143; *p* = 0.405; incremental AUC: *p* = 141, *p* = 0.413). There was no significant association between chromogranin expression and BMI among all patients with obesity (*r* = 0.222, *p* = 0.223), among patients with T2D (*r* = 0.23, *p* = 0.926), or those without T2D (*r* = 0.225, *p* = 0.459). However, among patients with T2D, the numbers of chromogranin‐expressing cells correlated with plasma glucose in the liquid meal test (60 min: *r* = 0.509; *p* 0.031; 120 min: *r* = 0.517, *p* = 0.031, glucose AUC *r* = 0.513, *p* = 0.031; incremental AUC *r* = 0.705, *p* = 0.004). In patients without T2D and in controls, there were no significant correlations between chromogranin and the markers of glucose homeostasis (controls: glucose AUC 0.143, *p* = 0.787, glucose incremental *r* = −0.314, *p* = 0.544, insulin AUC *r* = −143, *p* = 0.797; insulin AUC incremental *r* = −0.143, *p* = 0.797; patients no T2D: glucose AUC *r* = −0.140, *p* = 0.665; glucose incremental AUC *r* = −0.252, *p* = 0.430; insulin AUC *r* = −0.357, *p* = 0.255; insulin incremental AUC *r* = −0.308, *p* = 0.331).

In comparison of patients with prediabetes (*n* = 3) with the other patients with obesity without T2D (*n* = 12) no differences were seen in the numbers of immunopositive cells (chromogranin A, GLP‐1, or serotonin) or NmU areas. Similarly, in the T2D group, metformin (*n* = 17) or metformin in combination with GLP‐1 analogs (*n* = 1) or DPP4 (*n* = 9), no differences were seen in the cell numbers as compared with the nonusers.

### 3.2 GLP‐1

GLP‐1 expression was present in duodenal mucosa in a subpopulation (about 0.6%) of villous and crypt epithelial cells (Figure 2), showing cytoplasmic expression. In two patients with T2D and in one patient without T2D, immunohistochemical staining or scanning was technically unsatisfactory and could not be analyzed. There were no sex‐related differences. In comparison to controls, the proportion of GLP‐1 expressing cells was significantly higher in all patients (*p* = 0.006) and similarly in the subgroup of patients with T2D (*p* = 0.006; Figure 2). The subgroup of non‐T2D patients showed a trend for a higher proportion of GLP‐1 expressing cells (*p* = 0.07). No difference was seen between patients with or without T2D (*p* = 0.165).

Proportion of GLP‐1 expressing cells showed a positive correlation with blood HbA1C concentration (Figure 3; all subjects including controls *r* = 0.454, *p* = 0.005; all patients *r* = 0.443, *p* = 0.015; T2D patients *r* = 0.580, *p* = 0.014; patients without T2D *r* = 0.281, *p* = 0.331; controls *r* = 0.359, *p* = 0.553), and with plasma glucose concentration during meal test in all subjects (0 min: *r* = 0.379, *p* = 0.028; 30 min: *r* = 0.382, *p* = 0.028; 60 min: *r* = 0.487, *p* = 0.018; 120 min: *r* = 0.392, *p* = 0.028, AUC *r* = 0.455, *p* = 0.0018; incremental AUC *r* = 0.442, *p* = 0.018). In all patients with obesity, glucose levels during meal test correlated significantly with duodenal GLP‐1 cell numbers (0 min: *r* = 0.388, *p* = 0.048; 30 min: *r* = 0.330, *p* = 0.086; 60 min: *r* = 0.456, *p* = 0.048; 120 min: *r* = 0.370, *p* = 0.058, AUC *r* = 0.405, *p* = 0.048; incremental AUC *r* = 0.399, *p* = 0.048), but among the subgroups of patients with or without T2D or in the controls, no significant correlations emerged. No significant correlation with serum insulin concentration during meal test (time points 0 h, 30 min, 60 min, and 120 min; AUC or incremental AUC) was seen in the whole case series or in any subgroup (all: 0 min, *r* = 0.147, *p* = 0.392; 30 min *r* = 0.71, *p* = 0.694; 60 min *r* = 0.157, *p* = 0.376, 120 min *r* = 0.231, *p* = 0.189, AUC *r* = −0.104, *p* = 0.590; incr. AUC *r* = −109, *p* = 0.595; all patients: 0 min *r* = −127, *p* = 0.496; 30 min *r* = −3193, *p* = 0.325; 60 min *r* = −0.063, *p* = 0.745; 120 min *r* = 0.58, *p* = 0.766; AUC *r* = 0.144, *p* = 0.416; incr. AUC *r* = 0.119, *p* = 0.503; patients T2D: *r* = −0.213, *p* = 0.412; 30 min *r* = −0.333, *p* = 0.208; 60 min *r* = −0.183, *p* = 0,498; 120 min *r* = −179, *p* = 0.506; AUC *r* = −0.241, *p* = 0368; incr. AUC *r* = −0.247, 0.356; patients, no T2D: 0 min *r* = −0.029, *p* = 0.923; 30 min *r* = −0.84, *p* = 0.795, 60 min *r* = −0.071, *p* = 0.817; 120 min *r* = 0.154, *p* = 0.616, AUC *r* = −0.060, *p* = 0.845, incr. AUC *r* = −0.011, *p* = 0.972; controls 0 min *r* = −821, *p* = 0.089; 30 min *r* = 0.1, *p* = 0.873; 60 min *r* = 0.5, *p* = 0.391; 120 min *r* = −0.6, *p* = 285; AUC *r* = −100, *p* = 873; incr. AUC *r* = −0.100, *p* = 0.873). The proportion of GLP‐1 expressing cells did not correlate with BMI (all *r* = 202; *p* = 0.237; controls *r* = −0.600, *p* = 285; all patients *r* = −116, *p* = 0.533; T2D *r* = −248, *p* = 0.338, patients, no T2D: *r* = 0.231, *p* = 0.427).

### 3.3 Serotonin

Serotonin (5‐HT) expression was present in a subpopulation (about 1.6%) of duodenal villus epithelial cells (Figure 4), the proportion of serotonin positive cells being significantly higher in patients as compared to controls (controls vs. all patients *p* = 0.013; controls vs. non‐T2D patients, *p* = 0.027; controls vs. T2D patients *p* = 0.06; Figure 4). No difference was detected between the sexes or the patients with or without T2D. The proportion of serotonin expressing cells showed a trend for positive correlation with BMI among all subjects (*r* = 0.297, *p* = 0.066), but among patients no association was seen. There were no significant correlations between serotonin expression and glucose or insulin levels during meal test.

There were nine subjects using antidepressant medication (median age, 45.3 years, range 24.2–57.0; all female), four among patients with T2D, 4 among patients without T2D, and one among the controls. The proportion of serotonin‐expressing cells did not significantly differ between the antidepressant medication users (*n* = 9; median 1.8%; range 0.41%–2.46%) as compared with the nonusers among patients and controls (*n* = 27; 1.5%; 0.55%–2.61%; *p* = 0.136, Mann–Whitney). Since the high proportion of serotonin‐expressing cells was associated with obesity (see above) the association of the antidepressant drug use was separately assessed among patients with obesity. Among all patients with obesity, there was a trend (*p* = 0.063) for higher serotonin cell numbers in antidepressant users (*n* = 8; 1.9: 1.54%–2.46%) as compared with the nonusers (*n* = 22; 1.56%: 0.71%–2.61%). Among patients with T2D, the difference was significant (users: *n* = 4; 1.93%: 1.54%–2.46%; nonusers, *n* = 14; 1.56%: 0.71%–2.21%; *p* = 0.008), but among patients without T2D, no significant differences emerged (users *n* = 4; 1.75%: 1.54%–2.07%; nonusers *n* = 8; 1.6%: 1.23%–2.63%; *p* = 0.808). Use of antidepressant drugs was not associated with BMI in any group. For chromogranin A or GLP‐1, no association with antidepressant medication was seen (data not shown).

### 3.4 NmU

**FIGURE 5:**
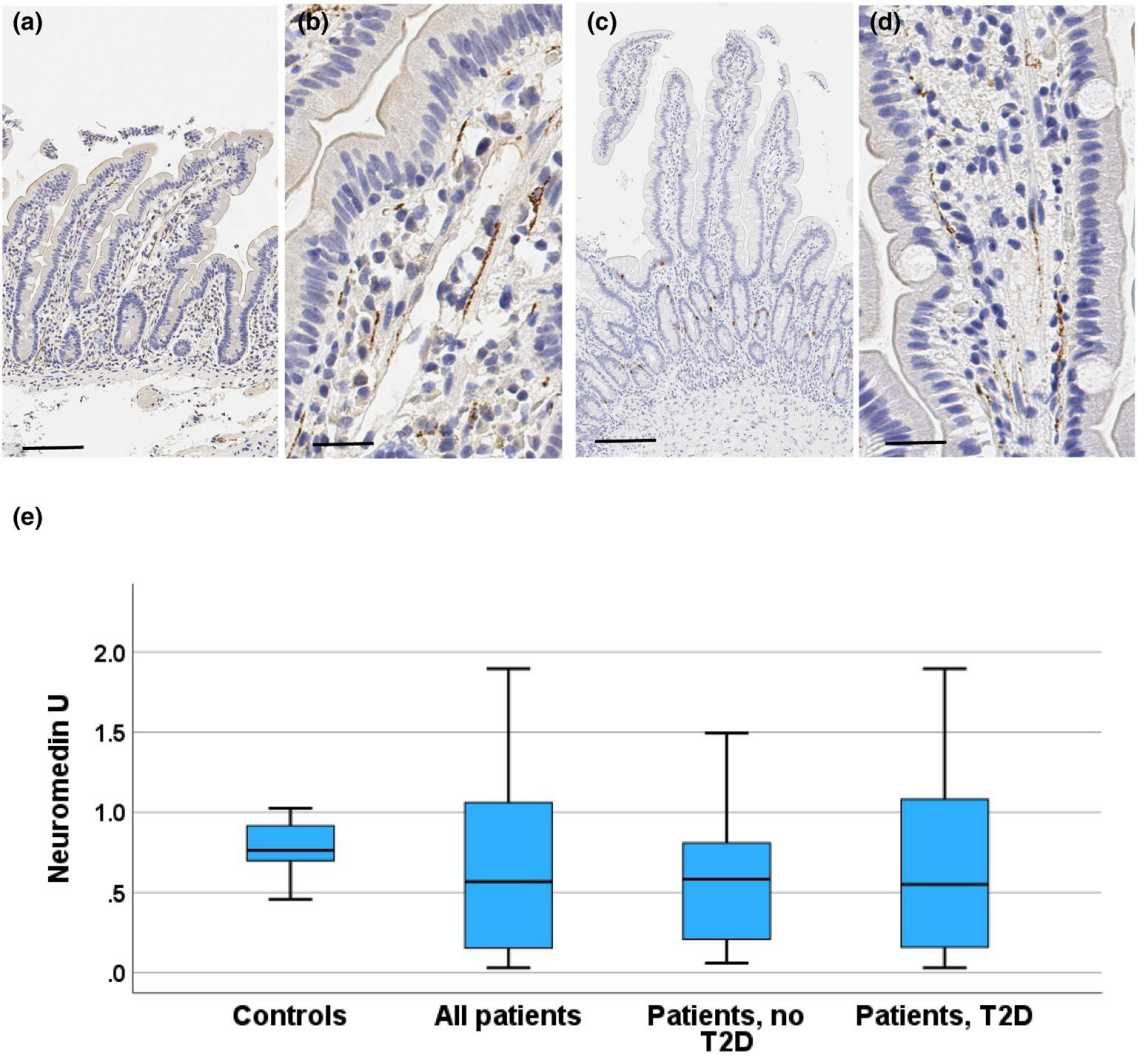
Neuromedin U. Photomicrographs from duodenal biopsies stained for Neuromedin U (a, b, control subject; c, d patient with T2D), and a boxblot (e) showing the distribution of Neuromedin U expression (proportion of lamina propria area). Images with a low (a, c; reference line = 100 μm) and a high (b, d; reference line = 20 μm) magnification are shown. Positively stained (brown) fiber‐like areas are seen in the lamina, mostly oriented with the long axis of the villi. In the boxplot, horizontal lines indicate median, boxes 75% percentiles, and whiskers show the range. There were no significant differences between the groups.

NmU expression was seen in all cases and located in lamina propria, where positive staining formed thin ribbons, likely following the course of autonomous nerve fibers (Figure 5). The areal proportion of NmU staining in the duodenal lamina propria ranged between 0.028% and 1.89% (median 0.61, mean 0.64). There were no significant differences between the groups (Figure 5). We did not see any significant relationship with BMI, glucose, or serum insulin levels among all subjects, patients with T2D, or controls (data not shown). There was a weak positive association between NmU expression and insulin levels during the liquid meal test (all subjects: incremental AUC, *r* = 0.363, *p* = 0.045; patients without T2D: incremental AUC *r* = 0.794, *p* = 0.024; 60 min *r* = 0.709, *p* = 0.044; 120 min *r* = 0.661, *p* = 0.045).

### 3.5 Correlation of duodenal chromogranin A, GLP‐1, serotonin, and NmU expression

Proportion of chromogranin expressing cells showed a significant correlation with the proportions of serotonin (*r* = 0.475 *p* = 0.003) or GLP‐1 expressing cells (*r* = 0.486, *p* = 0.003). For NmU, no significant correlations appeared.

### 3.6 Ratios of specific subpopulations of neuroendocrine cells and chromogranin A expressing cells

**FIGURE 6:**
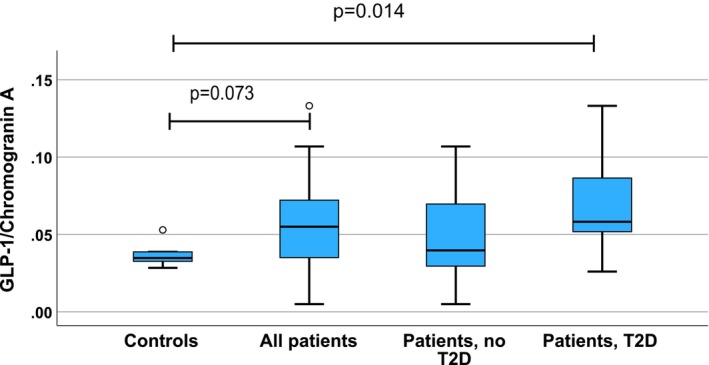
Box plot showing the ratios of percentages of GLP‐1 and chromogranin A expressing cells in duodenal mucosa in different groups of subjects. In the boxplot, horizontal lines indicate median, boxes 75% percentiles, and whiskers show the range. Controls and all patients were compared with the Mann–Whitney test. The controls, patients without and with T2D were compared with the Kruskal–Wallis test followed by groupwise post hoc analysis with the Dunn's tests, and adjustment for multiple comparisons with the Benjamini–Hochberg procedure. No difference was seen between controls and patients without T2D (*p* = 0.430) or between patients with or without T2D (*p* = 0.107; Mann–Whitney *U* test).

Since chromogranin A labels most of the intestinal neuroendocrine cells, we were interested in assessing whether the ratios of the GLP‐1 and serotonin expressing cells with the chromogranin A expressing cells are related to obesity or T2D. As compared with the controls, the ratio of GLP‐1 expressing cells to chromogranin A expressing cells tended to be higher among all patients (*p* = 0.073) and was higher in the patients with T2D (*p* = 0.014), but not among the non‐T2D patients (*p* = 0.430; Mann–Whitney *U* test; Figure 6) as compared with the controls. These findings suggest that the relative proportion of GLP‐1 expressing cells of all neuroendocrine cells is increased in T2D patients. However, the ratio did not differ between patients with and without T2D (*p* = 0.107). Among all patients with obesity, the GLP‐1/chromogranin A ratio correlated with meal test blood glucose at 60 min (*r* = 0.438, *p* = 0.047), glucose AUC (0.379, *p* = 0.047), glucose incremental AUC (0.398, *p* = 0.047), and HbA1C (*r* = 0.426, *p* = 0.019; Spearman), but among subgroups of the patients, no significant associations were seen.

Interestingly, in the controls, the GLP‐1/chromogranin A ratio showed a negative correlation with blood glucose in the meal test at 30, 60, and 120 min (*r* = −1, *p* = 0.002; *r* = −0.9, *p* = 0.037; *r* = −0.9, *p* = 0.037, respectively), glucose AUC (*r* = −1, *p* < 0.002), blood insulin at 120 min (−0.900, *p* = 0.037), and a negative correlation with BMI (−0.9, *p* = 0.037). There was no association with Hb1AC. The ratio of serotonin and chromogranin A expressing cells did not show any significant difference between the groups or any significant correlations with the markers of glucose homeostasis.

## 4 DISCUSSION

We report here that obesity is associated with an increase of duodenal chromogranin A, GLP‐1, and serotonin‐expressing cells. Among patients with obesity, T2D status did not affect the numbers of these cells. Unexpectedly, GLP‐1 cell numbers correlated positively with the signs of hyperglycemia, such as HbA1C concentration and glucose concentration during the meal test, but no association with insulin response was seen. These findings show that obesity is associated with hyperplasia of the duodenal endocrine cells but also suggest that such hyperplastic response may be associated with a relative insufficiency of the incretin response.

Chromogranin A, a component of neuroendocrine granules, is a general marker of intestinal neuroendocrine cells (Facer et al., 1985; Mouland et al., 1994) and may have a regulatory role in metabolism (Garg et al., 2023) Numbers of chromogranin A expressing cells were about 40% higher in patients with obesity as compared with the controls. The increase was similar in patients with and without T2D (Figure 1) and the cell numbers correlated positively with BMI among the whole study population. As summarized in Table 1, earlier studies have found that in the small intestine, the numbers of chromogranin A expressing cells are decreased in T2D (Jorsal et al., 2018) and obesity (Wölnerhanssen et al., 2017). Reasons for these discrepant findings are not clear and could be related either to staining and quantitation methods or differences in the populations studied, or both. Earlier studies used different antibodies for chromogranin A and manual quantitation of the positive cells, while we used an image analysis software.

Numbers of duodenal GLP‐1 expressing cells were about 107% higher in patients with obesity as compared to controls (Figure 2). In comparison of diabetic and nondiabetic patients, no significant differences appeared, a finding which agrees with Jorsal et al. (Jorsal et al., 2018), but contradicts the observations of Osinski et al. (2021), who reported that patients with obesity and T2D have about 44% less GLP‐1 expressing cells in the jejunum than patients with obesity but without T2D. In comparison with normal weight controls, previous studies on duodenal GLP‐1 cell numbers in T2D or obesity are not conclusive, as both increase (Theodorakis et al., 2006), no differences (Jorsal et al., 2018) or decrease (Wölnerhanssen et al., 2017) have been reported (Table 1). Osinski et al. found an overall decrease of jejunal GLP‐1 secreting cells in obesity, but subjects in their lean control group were older and composed of patients with gastric or pancreatic tumor and signs of hepatic and systemic inflammation, these conditions possibly affecting the intestinal endocrine cells. As already discussed for chromogranin A expression patterns, differences in methods and the composition of the cohorts in terms of severity of obesity and dysglycemia may explain the discrepant observations. In addition, nutritional differences may explain some divergent observations. High‐fat diet in both patients with obesity (Aranias et al., 2015) and in mice (Aranias et al., 2015; Chen et al., 2023) is associated with increased numbers of GLP‐1 expressing cells.

We were not able to measure GLP‐1 concentrations in the meal test, and the functional consequences of increased GLP‐1 cell numbers in persons with obesity remain speculative. Considering the relationship of the observed GLP‐1 cell hyperplasia in obesity and the incretin response, it is quite unexpected that the numbers of duodenal GLP‐1 expressing cells correlated positively with markers of dysglycemia including HbA1C concentration (Figure 6) and poor glucose tolerance during the meal test. These findings were evident both among the group consisting of all subjects and among patients with obesity and suggest that in patients with obesity, a gradual increase of GLP‐1 cells is associated with a decreasing incretin response as manifested by worsening dysglycemia. We saw no association between GLP‐1 cell numbers and serum insulin levels in the meal test, suggesting that there was no direct relationship between intestinal GLP‐1 cell numbers and insulin secretion. This supports the concept that the observed hyperplasia of GLP‐1 secreting cells in obesity with or without T2D is not associated with increased secretion of GLP‐1. However, since we did not measure blood GLP‐1 concentrations, the pathophysiological relationships between the numbers of duodenal GLP‐1 expressing cells, the actual secretion of GLP‐1, and the functional incretin response are not clear. In addition to insufficient secretion of GLP‐1, other mechanisms like degradation of GLP‐1, emergence of insufficient insulin production along with development of beta cell dysfunction, insulin resistance, or other factors modulating the incretin effect might explain the absence of correlation between GLP‐1 cell number and insulin concentration. Finally, there is some controversy on the relative importance of gut‐ versus pancreas‐derived GLP‐1 secretion in the incretin response (Chambers et al., 2017; Song et al., 2019).

Mechanism for the association of GLP‐1 cell abundance and the signs of inadequate incretin response in patients with obesity, and the dynamics of these abnormalities remain speculative. In mice models, high‐fat diet induced hyperplasia of GLP‐1 secreting cells was associated with no change (Hunt et al., 2022), increase, (Aranias et al., 2015) or decrease of GLP‐1 secretion (Chen et al., 2023), while in rats, hyperplasia of duodenal GLP‐1 secreting cells induced by a high fat diet associated with a decreased GLP‐1 response (Gniuli et al., 2010) indicating that in experimental pathological conditions GLP‐1 hyperplasia may be associated with decreased secretion. Theodrakis (Theodorakis et al., 2006) found that in newly diagnosed patients with T2D and BMI of 29 kg/m^2^ on average, the increased numbers of GLP‐1 secreting cells in the duodenum are present and associated with increased plasma GLP‐1 levels and increased insulin response. Our patients with T2D had more severe diabetes as shown by higher HbA1C concentration as compared with patients studied by Theodorakis et al. (2006), and even the patients without T2D had often abnormal HbA1C concentration. These differences suggest that our patients represented more advanced and likely a more long‐term dysglycemia. Collectively, these findings suggest that numerical and functional aberrations on intestinal GLP‐1 secreting cells may be diverse and depend on nutrition, the duration and severity of dysglycemia, and severity of other factors involved, such as the condition of islet beta‐cells and the severity of obesity. Accordingly, aberrations might include early hyperplastic response of GLP‐1 secreting cells associated with increased secretion, developing along with worsening of obesity, islet cell function, and insulin resistance to a later hyperplastic response associating with gradually worsening incretin effect.

Numbers of serotonin expressing cells were higher in patients without significant difference between T2D and nondiabetic patients. Similar findings in patients with obesity have been reported previously (Young et al., 2018). Serotonin expression showed a trend for a positive correlation (*p* = 0.066) with BMI, but no association with aberration of glucose homeostasis was detected. Mechanisms linking obesity and higher gut serotonin expressing cell numbers are unknown. However, serotonin has several effects on metabolism, including insulin secretion (Zhang et al., 2013). The prevalence of depression in obesity is reported to be high (Fulton et al., 2022). In our study, duodenal serotonin expression showed a trend for an increase in subjects with obesity using anti‐depressive medication as compared with the nonusers (*p* = 0.063), and in comparison of patients with T2D the difference was significant (*p* = 0.008). This association in T2D should be confirmed with a larger case series. Although mechanisms are not clear, our observation may support the hypothesis that aberration of duodenal endocrine function in association with obesity may have links with depression.

NmU is a neuropeptide with a suspected but not proven role as a decretin in rodents (Alfa et al., 2015; Kuhre et al., 2019) and roles in the regulation of metabolism and inflammation (Teranishi & Hanada, 2021). There are no previous studies about NmU in the human intestine. We found that NmU expression shows distribution in duodenal lamina propria reminiscent of that of autonomous nerve fibers. NmU was present in all cases with a similar pattern. Our studies did not show any indication for NmU to act as a decretin (Alfa et al., 2015) in humans, as a weak positive correlation between NmU area and serum insulin concentration in the meal test was detected, and no differences between the groups emerged. Serotonin has been implicated in the regulation of NmU expression (Nakashima et al., 2010), but the absence of correlation between serotonin and NmU expression in our study does not support a regulatory link locally in the mucosa.

Human intestinal neuroendocrine cells are known to differentiate from intestinal stem cells, with the process being regulated by several transcription factors (Zeve et al., 2022). There is some evidence that the regulation of differentiation is abnormal in obesity (Wölnerhanssen et al., 2017), but mechanisms are not yet known. In our study cohort, the numbers of cells expressing various endocrine markers all demonstrated proportionally quite similar increases in patients with obesity. In addition, we detected positive mutual correlations, as the chromogranin A expressing cell counts correlated with serotonin and GLP‐1 cell counts. These findings in obesity with or without T2D suggest that the cell types studied share factors regulating their numbers.

The proportion of chromogranin A expressing cells was about 20 times higher than that for GLP‐1 and about 7 times higher than that for serotonin. It has been suggested previously that chromogranin A labels the majority, but not all of the cells with known endocrine function in the intestinal epithelium, such as GLP‐1 positive cells (Facer et al., 1985; Jorsal et al., 2018; Kampmann et al., 2016). Interestingly, the ratio of GLP‐1 expressing cells to chromogranin A expressing cells was significantly higher in patients with T2D, but not in non‐T2D patients (Figure 6), suggesting that in addition to endocrine cell hyperplasia, obesity with T2D is associated with quantitatively abnormal differentiation patterns of the endocrine cells. In addition, we saw positive correlations between the GLP‐1/chromogranin A ratio and the determinants of defective glucose homeostasis, such as HbA1C and glucose AUC of the meal test among patients with obesity, but a negative correlation with blood glucose and insulin levels in the controls. These findings suggest that conditions associated with obesity, such as hyperglycemia or nutritional factors, might change the differentiation patterns of the intestinal endocrine cells, increasing the proportion of GLP‐1 cells. Considering hyperglycemia, such hyperplasia might be considered a physiologically relevant reaction as an attempt to increase the incretin response. The functional response may not be adequate. In islet beta cells, hypothetical dedifferentiation has been suggested to occur where exhausted cells lose secretion (Mezza et al., 2019). Clearly, mechanistic studies on the roles of dietary factors, obesity, and hyperglycemia in intestinal endocrine cell proliferation, differentiation, and function are called for.

We used immunohistochemistry with well‐controlled methods and widely used and reliable commercial antibodies to assess the duodenal samples. For quantitation, we used the image analysis program (Qupath) (Bankhead et al., 2017) able to recognize positive and negative epithelial cells and quantitate positively stained lamina propria areas in NmU stainings, thereby allowing an objective and unbiased quantification of the cells. In our study, the number of control subjects was low. However, the variation of the measurement results among the controls was clearly lower than in the patient groups. Our patients represent subjects with class 2 and 3 obesity, and therefore our observations cannot be generalized to represent T2D or obesity in general. Considering physiologically much higher numbers of GLP‐1 secreting cells in the more distal small intestine and colon, it would be important to analyze how their numbers are altered in severe obesity to obtain a complete picture of obesity‐related neuroendocrine cell changes in the intestinal mucosa.

In conclusion, our study shows that class 2 and 3 obesity with or without T2D is associated with an increase in duodenal chromogranin A, GLP‐1, and serotonin‐expressing cells. The number of GLP‐1‐expressing cells correlated positively with HbA1c and hyperglycemia during the meal test, suggesting that GLP‐1 cell hyperplasia is associated with an insufficient incretin effect. It remains to be studied which component of the incretin response is mainly involved. Finally, we demonstrated that NmU is constantly expressed in human duodenal lamina propria, but there was no evidence for NmU to act as a decretin.

## AUTHOR CONTRIBUTIONS

JH, MJS, VK, and TJK conceived and designed research; HB, JH, and TJK performed experiments, analyzed data, and interpreted results of experiments; HB and TJK prepared figures and drafted manuscript; all authors edited and revised manuscript, and approved final version of manuscript.

## FUNDING INFORMATION

J.H. was supported by the Finnish Foundation for Cardiovascular Research, the Northern Finland Health Care Support Foundation, Finnish Government Grants for Health Research, and the Finnish Medical Foundation.

## CONFLICT OF INTEREST STATEMENT

The authors declare no competing interests.

## ETHICS STATEMENT

All subjects gave written permission to take part in the study, and the Ethics Committee of the Northern Ostrobothnia Hospital District approved the study design.

## Data Availability

The data cannot be shared at this time due to legal/ethical reasons.
